# Impact of problematic substance use and social anxiety on school functioning: Insights from the Texas Youth Depression and Suicide Research Network

**DOI:** 10.1371/journal.pmen.0000621

**Published:** 2026-07-22

**Authors:** Regina Baronia, Brianna L. Minshall, Abu Minhajuddin, Lynnel C. Goodman, Elise N. Marino, Donald Egan, Gabrielle M. Armstrong, Denise Baughn, Anuththara H. Lokubandara, Sarah L. Martin, Jair C. Soares, Sarah M. Wakefield, Madhukar H. Trivedi

**Affiliations:** 1 Department of Psychiatry, Texas Tech University Health Sciences Center, Lubbock, Texas, United States of America; 2 Department of Psychiatry, Center for Depression Research and Clinical Care, Peter O’Donnell Jr. Brain Institute, University of Texas Southwestern Medical Center, Dallas, Texas, United States of America; 3 Peter O’Donnell Jr. School of Public Health, University of Texas Southwestern Medical Center, Dallas, Texas, United States of America; 4 Department of Psychiatry and Behavioral Sciences, Be Well Institute on Substance Use and Related Disorders, University of Texas Health Science Center at San Antonio, San Antonio, Texas, United States of America; 5 Menninger Department of Psychiatry and Behavioral Sciences, Baylor College of Medicine, Houston, Texas, United States of America; 6 Department of Psychiatry and Behavioral Sciences, University of Texas Medical Branch, Galveston, Texas, United States of America; 7 Department of Psychiatry, Texas Tech University Health Sciences Center, El Paso, Texas, United States of America; 8 Louis A. Faillace Department of Psychiatry and Behavioral Health, The University of Texas (UT Health) at Houston, Houston, Texas, United States of America; Baylor Scott and White Research Institute, UNITED STATES OF AMERICA

## Abstract

Early onset substance use and social anxiety are associated with adverse psychosocial outcomes, including impaired academic performance. This study aimed to investigate if problematic substance use is associated with poorer school functioning and if social anxiety is associated with this relationship. Participants (n = 711), aged 8–20, were enrolled in the Texas Youth Depression and Suicide Research Network registry study aiming to characterize youth with depression and/or suicidality. Baseline data from CRAFFT 2.1 + N, MINI-KID, SAS-SR, and PHQ-A were used for multivariate analyses using linear regression to examine the relationship between problematic substance use and school functioning, as well as the interaction between problematic substance use and social anxiety and their association with school functioning. Over 20% of participants met criteria for problematic substance use, with alcohol and marijuana being the two most common reported substances used. Youth with problematic substance use were on average two years older and had significantly greater impairment in school functioning compared to youth without problematic substance use. However, youth with problematic substance use and social anxiety had comparable impairments in school functioning compared to those with problematic substance use that did not have social anxiety. In youth without problematic substance use, having social anxiety was associated with similar levels of impairment in school functioning compared to youth with problematic substance use alone. The significant co-occurrence of problematic substance use, social anxiety, and depression suggests the need for early identification and intervention to prevent and/or mitigate impairment in school functioning. Significant levels of comorbid depression and/or suicidality experienced by our participants may have impacted our findings. Future research comparing youth with and without depression will help elucidate this.

## Introduction

Substance use among youth is a growing public health concern, often leading to the development of a substance use disorder (SUD) later in life [1]. Notably, approximately 16% of adolescents meet diagnostic criteria for alcohol use disorder and/or cannabis use disorder [2]. Early initiation of substance use is associated with anxiety, depression, and an increased risk of suicide [3–6]. Further, a previous study showed depression and suicidality in youth were predictive of multiple substance use in youth [7]. Together, these outcomes suggest a bidirectional relationship between depression, suicidality, and substance use.

Adolescents with persistent binge drinking and greater frequency of marijuana use have increased susceptibility to SUDs [8]. Adolescence is a pivotal phase of psychological, social, and neurological development. Substance use during this time can disrupt functioning across multiple domains, including school functioning and peer relationships, and heighten vulnerability to SUDs and other psychiatric disorders [9–11].

Evidence from epidemiologic and treatment studies suggest that problematic substance use and social anxiety are comorbid conditions, and that the interaction is variable [12]. Social anxiety disorder, characterized by excessive fear in social situations and heightened sensitivity to negative evaluation with resulting avoidance of social situations, [13], typically emerges around the age of 13 years, just as adolescents transition from familial dependence to becoming increasingly fixated on developing peer-focused relationships [14]. Challenges with this developmental transition, such as poor friendship quality, peer rejection, and peer victimization are associated with increased social anxiety [15].

Social anxiety has been associated with problematic substance use and impairments in school functioning [16]. A systematic review showed that youth with social anxiety disorder are more likely to use tobacco and marijuana [17], with studies suggesting substance use as a form of self-medication [18–20], and others reporting that youth may engage in substance use to manage subjective anxiety [21]. Individuals with social anxiety disorder are also less likely to achieve passing grades, qualify for vocational or academic programs, finish secondary education, or obtain a university degree [22].

**Fig 1:**
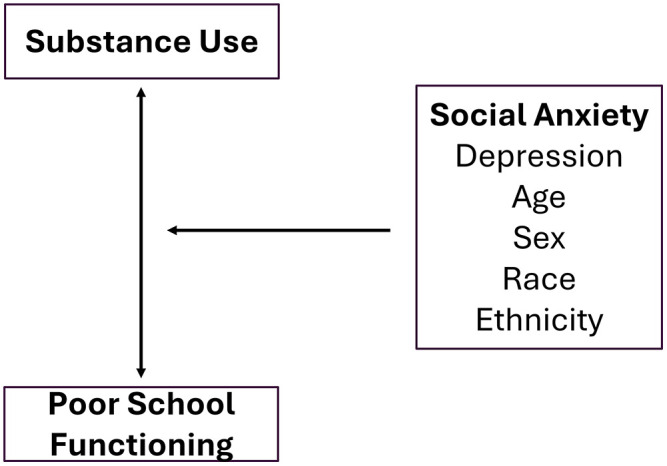
Conceptual framework for proposed associations in this study. Bolded factors indicate main variables of interest. Not bolded factors indicate covariates of interest.

Taken together, evidence suggests high comorbidity between social anxiety, substance use, and difficulties in school functioning. Therefore, the present study aimed to examine whether problematic substance use is associated with diminished school functioning among youth. Additionally, this study assessed whether the presence of social anxiety is associated with this relationship (see Fig 1). We hypothesized that youth with problematic substance use would demonstrate significantly poorer school functioning compared to those without problematic substance use, and that youth with both problematic substance use and social anxiety would exhibit the most severe impairments in school functioning.

## Method

### Ethics statement

This study was performed in line with the principles of the Declaration of Helsinki. The University of Texas Southwestern Medical Center Institutional Review Board (STU2020–0665) approved all procedures. All adult participants provided written consent after reviewing the Informed Consent Form. For minors, written consent was obtained from a parent, guardian, or legally authorized representative, and age-appropriate written assent was obtained from the participant. Study procedures were explained in a developmentally appropriate manner, and both consent and assent were required prior to participation.

### Reporting guidelines

This manuscript conformed to the STROBE Guidelines for observational studies.

### Participants

This work utilized data from the Texas Youth Depression and Suicide Research Network (TX-YDSRN), a multi-site, prospective, observational study examining the trajectories of youth to emerging adults with depression and/or suicidality. It was created as a part of the Texas Child Mental Health Care Consortium (TCMHCC) to establish a statewide research network across 12 Health-Related Academic Institutions in Texas. Participants between the ages of 8 and 20 were recruited from participating clinical subsites. Youth were eligible if they screened positive for depression, defined as a score ≥ 3 on the Patient Health Questionnaire-2 (PHQ-2) or ≥ 10 on the 9-item Patient Health Questionnaire Modified for Adolescents (PHQ-A), or for suicidality, defined as endorsement of suicidal ideation or behavior on the 16-item Concise Health Risk Tracking – Self Report (CHRT-SR_16_) or PHQ-A item 9. Further, youth currently receiving treatment for depression from participating clinics were also eligible to participate. Exclusion criteria included acute medical or psychological conditions that would make participation unsafe or unfeasible. The rationale and study design has been previously described [23]. For the current analyses, we focused on youth who were among the first 1,000 participants enrolled in the study (consented from August 26^th^, 2020, to March 22^nd^, 2022) and were also enrolled in school and had completed the Social Adjustment Scale – School Module (SAS-SR). This is a secondary analysis of a larger, on-going study. Based on a final sample size of 711, medium effects can be detected. A total of 289 participants were excluded from the analyses for not having a complete SAS-SR (*n* = 711).

### Measures

**Car, Relax, Alone, Forget, Friends, Trouble version 2.1 + Nicotine (CRAFFT 2.1 + N).** The CRAFFT 2.1 + N, [24], screens for substance use risk and problems in youth across six domains of functional impairment. The first four items refer to the number of days of use of alcohol, marijuana, other substances, and nicotine/tobacco use over the past 12 months. The remaining six items assess associated problems regarding substance use or consequences that yield the “CRAFFT” acronym. These include: riding in an automobile driven by someone under the influence of alcohol or drugs (“Car”); using substances to relax or fit in (“Relax”); using substances when alone (“Alone”); forgetting things when using substances (“Forget”); being told by family or friends to cut back on substance use (“Friends”); and getting into trouble when using substances (“Trouble”). The total score ranges from 0–6, and those scoring ≥ 2 were categorized as having problematic substance use and 0–1 as having no problematic substance use [25]. Psychometric properties have been demonstrated previously [24].

**Mini-International Neuropsychiatric Interview for Children and Adolescents (MINI-KID).** The MINI-KID [26] is a short, diagnostic interview for psychiatric disorders in children and adolescents administered by a clinician. A parent version is also used for minors to achieve the best estimate rating of diagnoses. For adult participants, the best estimate is based on the participant version only. The MINI-KID was used to identify participants with social anxiety disorder. Psychometrics among children and adolescents have been well-established [26].

**Social Adjustment Scale School Module – Self-Report (SAS-SR).** The SAS-SR School Module [27,28] is a 6-item measure that assesses school functioning and performance that is a sub-section of the 54-item Social Adjustment Scale. Higher scores indicate poorer school performance [29]. The version used in the study asked the participant about the number of class days missed, the ability to keep up with schoolwork, how often they have been ashamed of their schoolwork, arguments with people at school, feeling upset at school, and whether they found their schoolwork interesting. Numerous studies have reported psychometric properties [27,28].

**Patient Health Questionnaire Modified for Adolescents (PHQ-A).** The PHQ-A [30] is a 9-item self-report measure that assesses symptoms in all nine domains of a major depressive episode. Each item is scored from 0 (not at all) – 3 (nearly every day), for a total score ranging from 0-27. Symptom severity is divided into five categories: Minimal (0–4), Mild (5–9), Moderate (10–14), Moderately Severe (15–19), and Severe (20–27). Psychometric properties in primary care youth samples have been demonstrated previously [30].

### Data analysis

Bivariate analyses examining differences in demographic and clinical characteristics by problematic substance use and social anxiety, respectively, were conducted using *t*-tests for continuous variables and chi-squared tests for categorical variables. Multivariate analyses using linear regression were performed to examine the relationship between problematic substance use and school functioning and the interaction between problematic substance use and social anxiety regarding school functioning, controlling for age, sex, race, ethnicity, and depression severity. All analyses were completed using SAS version 9.4 (SAS Inc., Cary, NC). Significance was determined by *p*-values < .05.

## Results

Of the 711 participants, most of the sample was female (74.0%), White (67.5%), and non-Hispanic (53.9%) with a mean average age of 15.2 years (*SD* = 2.5). For substance use, 22.2% of youth met criteria for problematic substance use measured by CRAFFT 2.1 + N (*M* = 3.42, *SD* = 1.23 for youth with problematic substance use versus *M* = 0.17, *SD* = 0.37 for youth without problematic substance use). Older age was significantly associated with membership in the problematic substance use group *t*[283.9] = 8.65, *p* < .0001. Within the last 12 months, most youth (66.2%) reported using no substance. Alcohol use, at least once in the last 12 months, was reported by 25.6% of the sample along with 23.5% for marijuana use, 15.3% for tobacco use, and 5.8% for use of a different substance to get high.

Youth with problematic substance use had significantly greater impairment in school functioning compared to youth without problematic substance use (*t*[709] = 2.21, *p* = .03). There was no difference in PHQ-A scores between youth with and without problematic substance use. Descriptive data are detailed in Table 1.

**Table 1:** Comparison of demographics and clinical features of youth with and without problematic substance use.

	Total Sample n = 711	No substance use problem n = 553 (77.8%)	Substance use problem n = 158 (22.2%)	Χ^2^	*t*	*p*-value
**Sex, n (%)**				2.78		.10
Female	526 (74.0)	401 (72.5)	125 (79.1)			
Male	185 (26.0)	152 (27.5)	33 (20.9)			
**Race, n (%)**				6.18		.19
White	480 (67.5)	378 (68.4)	102 (64.6)			
Black/African American	66 (9.3)	54 (9.8)	12 (7.6)			
More Than One Race	78 (11.0)	59 (10.7)	19 (12.0)			
Other	77 (10.8)	57 (10.3)	20 (12.7)			
Unknown	10 (1.4)	5 (1.0)	5 (3.2)			
**Ethnicity, n (%)**				2.77		.25
Hispanic	321 (45.1)	241 (43.6)	80 (50.6)			
Non-Hispanic	383 (53.9)	307 (55.5)	76 (48.1)			
Unknown	7 (1.0)	5 (1.0)	2 (1.3)			
**Social Anxiety, n (%)**				4.50		**.03**
No	472 (66.4)	356 (64.4)	116 (73.4)			
Yes	239 (33.6)	197 (35.6)	42 (26.6)			
**Age at Consent, mean (SD)**	15.2 (2.5)	14.8 (2.5)	16.5 (2.2)		8.04	**<.0001**
**PHQ-A Total Score, mean (SD)**	12.6 (6.3)	12.4 (6.2)	13.4 (6.6)		1.83	.07
**SAS-SR School Module Score, mean (SD)**	2.44 (0.8)	2.4 (0.8)	2.6 (0.8)		2.21	**.03**
**ADHD (any type), n (%)**					0.07	.79
No	389 (54.7)	326 (59.0)	63 (39.9)			
Yes	322 (45.3)	227 (41.1)	95 (60.1)			

Note: PHQ-A = Patient Health Questionnaire Modified for Adolescents; SAS-SR = Social Adjustment Scale Self-Report; ADHD = Attention-Deficit/Hyperactivity Disorder; SD = Standard deviation. Significant p-values (p < .05) are depicted in **bold**.

**Fig 2:**
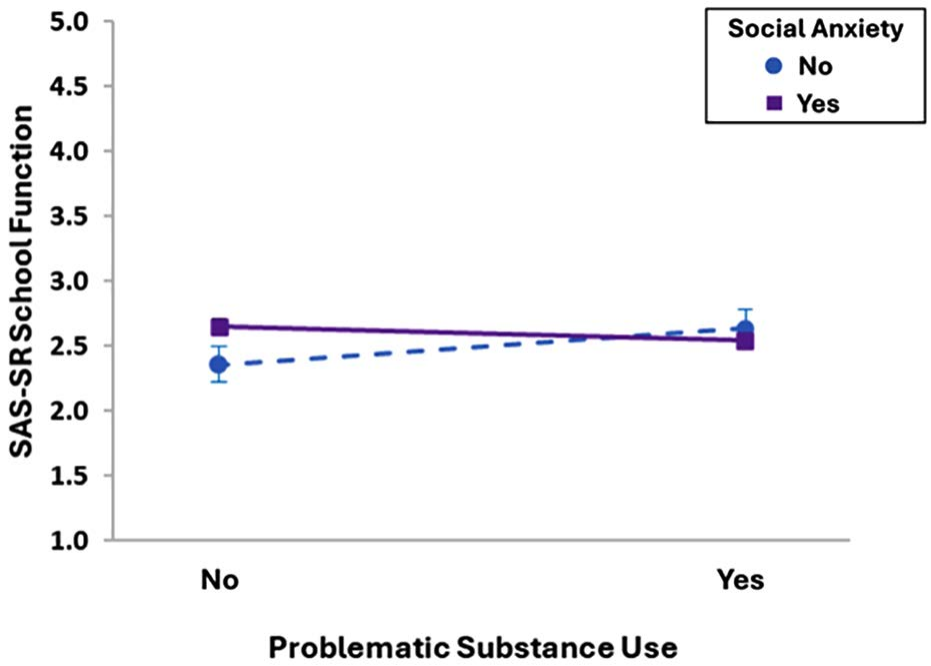
Interaction between problematic substance use and social anxiety with school functioning. Interaction plot showing the association of social anxiety with school functioning based on problematic substance use. Note: Problematic substance use is defined as mean CRAFFT2.1 + N score ≥ 2; CRAFFT2.1 + N = Car, Relax, Alone, Forget, Family, Trouble plus Nicotine; Social Anxiety was based on MINI-KID diagnosis; MINI-KID = Mini-International Neuropsychiatric Interview for Children and Adolescents; SAS-SR = Social Adjustment Scale Self-Report.

A sensitivity analysis was conducted to account for potential site effects. The number of participants with problematic substance use compared to those without did vary by site (*t*[11] = 25.08, *p* < .01). However, when added as a covariate into the interaction model, there was no significant main effect of site. As such, site was not included in the final model. When controlling for sex, age, race, ethnicity, ADHD diagnosis, and depression severity, the overall regression assessing the association between problematic substance use and/or social anxiety with school functioning was statistically significant (*R*^2^ = 0.29, *F*[9, 701] = 31.99, *p* < .0001; see Table 2). The interaction between problematic substance use and social anxiety was statistically significant (β = -0.38, *p* < .01). For youth with problematic substance use, also having social anxiety did not result in a significantly different association with impairments in school functioning compared to those without social anxiety. However, for youth without problematic substance use, those with social anxiety had greater impairment in their school functioning compared to those without social anxiety (see Fig 2).

**Table 2:** Linear regression main effects on school functioning.

Variable	β (SE)	*t*	*p*-value
Intercept	1.86 (0.18)	10.39	**<.0001**
Age at Consent	-0.02 (0.01)	-2.12	**.03**
Sex	0.01 (0.06)	0.22	.83
Race	-0.04 (0.05)	-0.67	.50
Ethnicity	-0.02 (0.05)	-0.37	.72
ADHD	0.18 (0.05)	3.47	**<.01**
PHQ-A Total Score	0.06 (0.004)	14.44	**<.0001**
Substance Use Problem	0.26 (0.07)	3.53	**<.001**
Social Anxiety	0.28 (0.06)	4.79	**<.0001**
Substance Use Problem * Social Anxiety Interaction	-0.38 (0.13)	-2.80	**<.01**
R^2^	0.29		
No. observations	711		

Note: ADHD = Attention-Deficit/Hyperactivity Disorder; PHQ-A = Patient Health Questionnaire Modified forAdolescents; SE = Standard error. Significant p-values (p < .05) are depicted in **bold**.

## Discussion

The present study examined whether problematic substance use is associated with impairments in school functioning at baseline. Further, we assessed whether the presence of social anxiety was associated with the relationship between problematic substance use and school functioning. Overall, our results showed more than a fifth of our sample met criteria for problematic substance use, problematic substance use and social anxiety interact to yield impairments in school functioning, and problematic substance use was associated with older age.

Our results demonstrated that over 20% of youth with depression and/or suicidality included in this study met criteria via the CRAFFT 2.1 + N for problematic substance use. This is comparable to observed national trends. The 2023 National Survey on Drug Use and Health reported that of all youth surveyed, 28.4% of youth aged 12–17 with a major depressive episode within the last 12 months used illicit drugs compared to 11.6% of youth aged 12–17 without a major depressive episode reporting illicit drug use [31]. In our sample, the most frequently used substances were alcohol and marijuana. While alcohol use may be influenced by accessibility, it is notable that recreational marijuana remains illegal in the state of Texas [32]. Nationally, in 2023, 14.6% of youth aged 12–20 years were past month alcohol users and 11.2% of youth aged 12–17 years reported using marijuana [31].

Contrary to a recent meta-analysis that suggested a positive association between depression, alcohol, marijuana, and tobacco use, in the present study problematic substance use was not associated with differences in depression symptoms compared to those without problematic substance use [3]. It is possible that variations in clinical diagnosis among studies (e.g., depression diagnosis versus depression symptoms) may have contributed to this difference. Additionally, results showed that youth with any type of ADHD diagnosis was associated with greater impairment in school functioning compared to youth without any ADHD diagnosis. This finding is consistent with other studies demonstrating that ADHD has a negative impact on school functioning (e.g., [33,34]).

Within our sample, we demonstrated that problematic substance use and social anxiety interact to contribute to impairments in school functioning. Inconsistent with our hypothesis, youth with problematic substance use who also had social anxiety did not differ from those without social anxiety with both groups showing similar impairment in school functioning. This unexpected finding raises questions about whether some youth may use substances as a form of self-medication for social anxiety, potentially masking some academic difficulties (e.g., self-medication hypothesis; [17,35]). It has been demonstrated that problematic substance use is negatively associated with school membership, increased odds of skipping school, getting lower grades, and lower academic engagement and higher dropout rates compared to students without problematic substance use [36–38]. Alternatively, it is possible that problematic substance use, alone, is associated with impairments in school functioning to a degree such that the presence of social anxiety does not yield a significant difference.

We also found that for youth without problematic substance use, having social anxiety resulted in greater impairment in school functioning compared to those without social anxiety. Previous work has shown that adolescents with anxiety disorders report increased feelings of impairment at school, have a higher risk of school refusal, and are less likely to seek higher education [17]. Youth with social anxiety may have difficulty with interpersonal relationships and this difficulty may be associated with impairments in school functioning [39].

Of clinical significance is the fact that the average age of participants with problematic substance use was approximately two years older than those without problematic substance use. This indicates that the time between the ages of fourteen and sixteen years may be a critical period for prevention and intervention efforts by parents and caregivers. Given the prevalence of substance use and social anxiety in our sample, prevention and intervention efforts should begin early, especially among those susceptible to depression and related symptoms. Preventive interventions that decrease substance use starts with the family and school, as these are the primary socializing environments. Further, primary care providers can play a vital role in informing universal prevention and screening in pediatric clinics [40]. Substance use in adolescence can impact school functioning, leading many school officials to frequently request clinicians for instruction in recognizing signs and symptoms and referral for treatment [41].

These results should be considered with some limitations in mind. First, data were not collected on the onset of substance use, the duration of substance use and social anxiety, conduct problems, cognitive ability, nor level of premorbid functioning. This information may have allowed stratification of participants based on duration and severity of either substance use or social anxiety and accounted for other individual factors like previous intervention. Second, not all the measures used are validated in the entire age range of our dataset. Validation papers are currently in preparation for measures used in TX-YDSRN that are not currently validated in the full age range of our dataset. Third, the binary nature of the social anxiety variable should be considered another limitation. It is possible that some youth did not meet diagnostic criteria for social anxiety disorder but may have had subclinical levels of social anxiety, thus diluting some effects. Fourth, the self-report nature of the SAS-SR is another limitation of the present study. In this clinical sample, there may have been some response bias (e.g., underreporting of achievements, viewing functioning more negatively) that should be taken into consideration when interpreting the results. Future studies should consider including objective school functioning data. Fifth, all participants were living in the state of Texas and must have been experiencing significant depression and/or suicidality to be included in the parent study. It is worth noting that our sample demographics map onto demographics of this age range in the state of Texas [42]. However, it is possible that our results may not generalize to nonclinical samples, male-dominated samples, or to youth not seeking care for depression and/or suicidality. Sixth, we were only able to capture substance use categorically for alcohol, marijuana, and tobacco, while all other substances were categorized as one group. Seventh, we did not have socioeconomic status data available for all participants. Future studies should investigate the association between environmental support and school functioning. Finally, this was an observational study with a cross-sectional design. As such, the direction of effects cannot be determined, and no causal inferences can be made.

Overall, the present study demonstrated that problematic substance use, alone, is associated with significant impairments in school functioning compared to youth without problematic substance use. Interestingly, for youth without problematic substance use, having social anxiety is associated with similar levels of impairment in school functioning compared to youth with problematic substance use alone. There exists a clinically significant intersection among problematic substance use, social anxiety, and impaired school functioning in youth with depression and/or suicidality. With the substantial amount of development occurring during this time, the co-occurrence of this constellation of symptoms is noteworthy. Early identification is key to improving the overall functioning of youth with this presentation, and likely will require an integrated prevention (e.g., combined cognitive-behavioral approaches targeting both social anxiety and substance use, school-based programs) and treatment approach that involves family, school, and primary care providers. Future studies should examine the temporal relationships between problematic substance use, social anxiety, and school functioning across multiple time points and look at these relationships in nonclinical samples to increase generalizability.
